# Earlier infantile immune maturation is related to higher DTP vaccine responses in children

**DOI:** 10.1038/cti.2016.7

**Published:** 2016-03-11

**Authors:** Anna Strömbeck, Anna-Carin Lundell, Inger Nordström, Kerstin Andersson, Ingegerd Adlerberth, Agnes E Wold, Anna Rudin

**Affiliations:** 1 Department of Rheumatology and Inflammation Research, Institute of Medicine, The Sahlgrenska Academy, University of Gothenburg, Gothenburg, Sweden; 2 Department of Clinical Bacteriology, Institute of Biomedicine, The Sahlgrenska Academy, University of Gothenburg, Gothenburg, Sweden

## Abstract

There are large inter-individual variations in vaccine-specific antibody responses in children. We sought to investigate whether early-life environmental factors and/or adaptive immune maturation were related to diphtheria–tetanus–pertussis (DTP) vaccine-specific antibody levels at 18 months of age. In the prospective FARMFLORA birth-cohort, including both farming and non-farming families, children were immunized with DTP vaccine at 3, 5 and 12 months of age. DTP vaccine-induced antibody levels were measured in plasma at 18 months of age. Infants' blood samples obtained at birth, 3–5 days, 4, 18 and 36 months and at 8 years of age were analyzed for total CD4^+^ T- and B-cell counts, proportions of naïve and memory T and B cells, and fractions of putative regulatory T cells by flow cytometry. Multivariate factor analysis was used to examine associations between immune variables and vaccine responses. The most apparent multivariate pattern was that higher anti-DTP antibody titers at 18 months of age were associated with lower infantile total counts of T and B cells in the blood. Furthermore, lower infantile total T- and B-cell blood counts were associated with higher proportions of circulating CD45RO^+^ memory T cells and to lower proportions of α4β7^+^ naïve T cells later in childhood. The multivariate findings were corroborated in univariate correlation analyses. Sex, delivery mode and dairy farm exposure were unrelated to the magnitude of DTP-specific antibody responses. Our results thus suggest that children with a more mature/activated infantile adaptive immunity respond with higher vaccine-induced anti-DTP antibody levels at 18 months of age.

In Sweden, the combination vaccine diphtheria–tetanus–acellular pertussis (DTP)–polio-*Haemophilus influenzae* type b is given initially at 3 months with boosters at 5 and 12 months and at 5–6 years of age. Vaccination of infants and young children is required to prevent infectious diseases in early life, but its effectiveness might be impeded by immaturity of the immune system (reviewed in PrabuDas *et al.*^1^). However, longitudinal studies investigating associations between the development/maturation progress of the adaptive immune system and magnitudes of DTP vaccine-induced antibody responses in children are lacking.

The adaptive immune system of newborns is essentially naïve, given the limited exposure to exogenous antigens *in utero*. In cord blood, naïve CD45RA^+^ and α4β7^+^ T cells constitute ~90% of the CD4^+^ T-cell pool.^2, 3, 4^ The proportions of these cells decrease significantly during the first 3 years in life, whereas proportions of CD45RO^+^ memory T cells concomitantly increases along with increased capacity to produce cytokines.^4, 5, 6^ Similarly, the proportion of immature/naïve CD5^+^ B cells decreases gradually from birth to adulthood, whereas the fraction of CD27^+^ memory B cells increases.^3, 7^ In parallel with postnatal B-cell maturation, there is an increase in IgG- and IgA-expressing B cells as well as total levels of these immunoglobulins in the circulation.^8, 9^

FOXP3^+^ regulatory T cells (Tregs) suppress proliferation and cytokine production of other T cells in response to both self- and microbial antigens.^10, 11, 12^ In adults, the majority of FOXP3^+^ Tregs express intracellular CTLA-4,^13^ which has a critical role for the suppressive mechanisms of these cells.^13^ Infants with higher proportions of circulating Tregs have lower fractions of memory T cells later in childhood.^4^ In mice, depletion of Tregs enhances vaccine-induced immunity.^14, 15^ However, the relation between proportions of blood Tregs and vaccine-induced antibody responses has not yet been studied in children.

Early-life environmental factors have been shown to influence adaptive immune maturation. The farming environment is rich in particles from microbes,^16, 17^ and growing up on a farm is associated with higher proportions of CD4^+^ memory T cells and with mononuclear cells more prone to produce mitogen-induced cytokines in the first 3 years of life.^18^ Further, the risk of developing allergy is lower among farmers' children compared with non-farmers' children.^18, 19, 20^

Contrary, children born by cesarean section have lower proportions of memory T cells in childhood.^21^ In addition, sex-related differences in vaccine-specific antibody levels have been reported, demonstrating that girls present with higher mumps and rubella antibody titers.^22, 23^ However, it remains to be elucidated whether these early-life environmental factors associate with the magnitude of DTP vaccine-induced antibody responses in children.

By studying the prospective FARMFLORA birth-cohort, in which 51 infants received the DTP vaccine at 3, 5 and 12 months of age, we sought to explore if early-life environmental factors and/or adaptive immune maturation before or in close proximity to immunization were related to DTP vaccine-specific antibody levels in plasma at 18 months of age. A principal finding was that children who displayed higher vaccine-induced anti-DTP antibody levels at 18 months of age had lower total numbers of peripheral T and B cells in the first 4 months of life. In addition, lower total numbers of peripheral T and B cells in infancy were associated with higher proportions of memory T cells later in childhood.

## Results

### Higher anti-DTP antibody titers are related to lower numbers of lymphocytes in infancy

**Figure 1:**
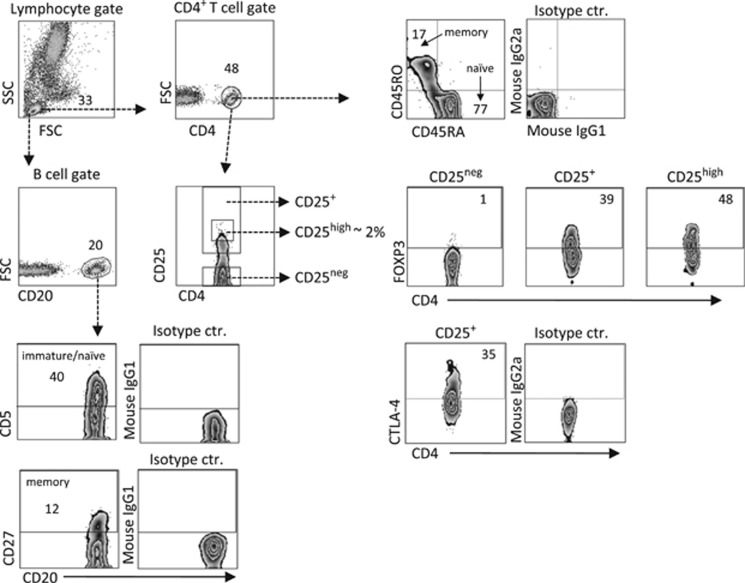
Representative flow cytometry plots showing our gating strategies in the FARMFLORA study. Lymphocytes were defined in forward (FSC) versus side scatter (SSC) plot. T and B cells were then gated according to their CD4 or CD20 expression, respectively. The CD45RO^+^CD45RA^-^ memory cells within the CD4^+^ T cells population were gated based on the isotype control. CD4^+^ T cells were also gated based on CD25^neg^, CD25^+^ or CD25^high^ expression. FOXP3^+^ CD25^high^ T cells were gated based on the lack of expression within the CD25^neg^ subset, and CTLA-4^+^CD25^+^ T cells were gated based on the isotype control (ctr.). The CD5^+^ immature/naive or CD27^+^ memory B-cell gate was based on respective isotype controls. Numbers represent the percentage of cells within the gate.

In the present study, all infants were immunized with the DTP vaccine at 3, 5 and 12 months of age. With the use of multivariate factor analysis, we first examined whether total CD4^+^ T- and B-cell counts, proportions of naïve and memory T and B cells, and/or fractions of putative Tregs in the blood at birth, 3–5 days and at 4 and 18 months were associated with specific DTP vaccine-induced antibody levels in plasma at 18 months of life. The gating strategy for the various T- and B-cell subsets assessed is presented in Figure 1.

**Figure 2:**
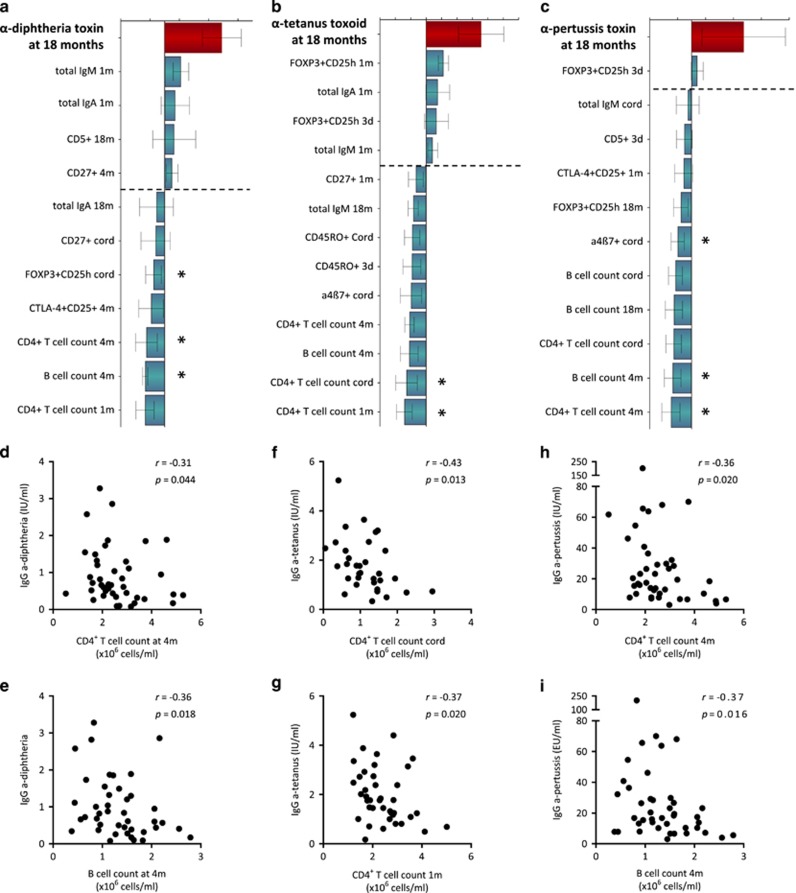
OPLS-loading column plot displaying the associations between *Y*, that is, post-vaccination plasma titers of (**a**) anti-diphtheria toxin antibodies, (**b**) anti-tetanus toxoid antibody titers and (**c**) anti-pertussis toxin antibody titers in plasma at 18 months of age. and *X* variables, that is, T- and B-cell variables over the first 18 months of life that characterize adaptive immune maturation. *X* variables (that is, immune parameters) with bars projected in the same direction as the *Y* variable (that is, the respective anti-DTP antibody titers) are positively associated, whereas parameters in the opposite direction are inversely related to the *Y* variable. The larger the bar and smaller the error bar, the stronger and more certain is the contribution to the model. VIP values are used to discriminate between important and unimportant predictors for the overall model. The OPLS-loading column plots are based on *X* variables with VIP values⩾1.25(**a**); 1.20 (**b**); 1.15 (**c**). The quality of the OPLS analyses was based on the parameters R2, that is, how well the variation of the variables is explained by the model, and Q2, that is, how well a variable can be predicted by the model. R2Y=0.25 (**a**); 0.30 (**b**); 0.29 (**c**). Q2=0.13 (**a**); 0.19 (**b**); 0.10 (**c**). Statistical significant correlations (Spearman's rank correlation test) between *Y* and *X* variables are indicated by asterisks in the respective OPLS analyses. A *P-*value⩽0.05 was regarded as being statistically significant (*P⩽0.05). (**d**–**i**) Representative univariate correlation plots from each OPLS analysis are shown below the respective loadings column plot.

Separate orthogonal projections to latent structure (OPLS) analyses for each toxin/toxoid were performed and are shown in Figures 2a–c. The final loading column plots display the immune parameters that associate most strongly, positively or negatively, with the vaccine-induced antibody levels in the respective models (Figures 2a–c). The most apparent pattern displayed for all three vaccines in the multivariate analyses was that higher vaccine-induced antibody levels at 18 months of age were negatively associated with total numbers of CD4^+^ T cells and B cells in the blood during the first 4 months of life (Figures 2a–c). Indeed, anti-diphtheria toxin (DT) antibody levels correlated negatively with the total counts of CD4^+^ T cells and B cells at 4 months of age (Figures 2d and e), as well as with the proportions of FOXP3^+^CD25^high^ cells of CD4^+^ T cells in cord blood (*r*=−0.37; *P*=0.03). Further, anti-tetanus toxoid (TT) antibody levels correlated negatively with total CD4^+^ T-cell counts in cord blood and at 1 month of age (Figures 2f and g). Similar to the DT vaccine, anti-pertussis toxin (PT) antibody levels correlated negatively with total CD4^+^ T-cell and B-cell counts at 4 months of age (Figures 2h and i), but also with the proportions of α4β7^+^ T cells in cord blood (*r*=−0.50; *P*=0.004). Levels of total IgM, IgA and IgG did not correlate with vaccine-induced anti-DTP antibody levels. These results indicate that children who have lower total numbers of peripheral T and B cells in early childhood are more likely to respond with higher anti-DTP antibody titers after vaccination.

### Higher total numbers of T and B cells in early infancy are related to lower proportions of memory T cells later in childhood

**Figure 3:**
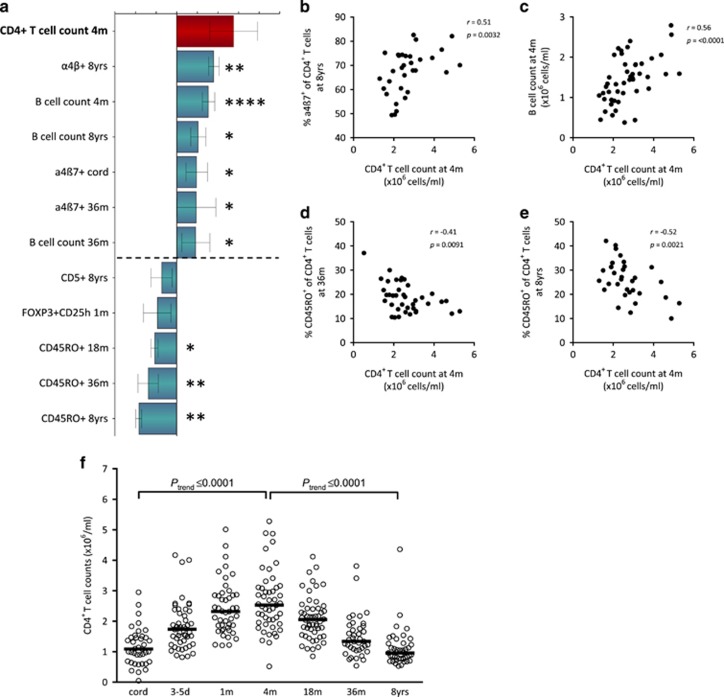
(**a**) OPLS-loading column plot displaying the associations between *Y*, that is, total numbers of CD4^+^ T cells at 4 months of age and *X* variables, that is, T- and B-cell variables over the first 9 years of life that characterize adaptive immune maturation. *X* variables with bars projected in the same direction as the respective *Y* variable are positively associated, whereas parameters in the opposite direction are inversely related to the *Y* variable. The larger the bar and smaller the error bar, the stronger and more certain is the contribution to the model. VIP values are used to discriminate between important and unimportant predictors for the overall model. The OPLS-loading column plot is based on *X* variables with VIP values⩾1.2. The quality of the OPLS analysis was based on the parameters R2, that is, how well the variation of the variables is explained by the model, and Q2, that is, how well a variable can be predicted by the model. R2Y= 0.52; Q2=0.49. Statistical significant correlations (Spearman's rank correlation test) between *Y* and *X* variables are indicated by asterisks in the OPLS analysis. A *P-*value⩽0.05 was regarded as being statistically significant (*P⩽0.05; **P⩽0.01; ***P⩽0.001 and ****P⩽0.0001). (**b**–**e**) Representative univariate correlation plots from the OPLS analysis. (**f**) Total numbers of blood CD4^+^ T cells within the lymphocyte gate at birth, 3–5 days, 1, 4, 18, 36 months and at 8 years of age. Horizontal bars indicate medians. ****P⩽0.0001, analysis of variance post-test for linear trend.

**Figure 4:**
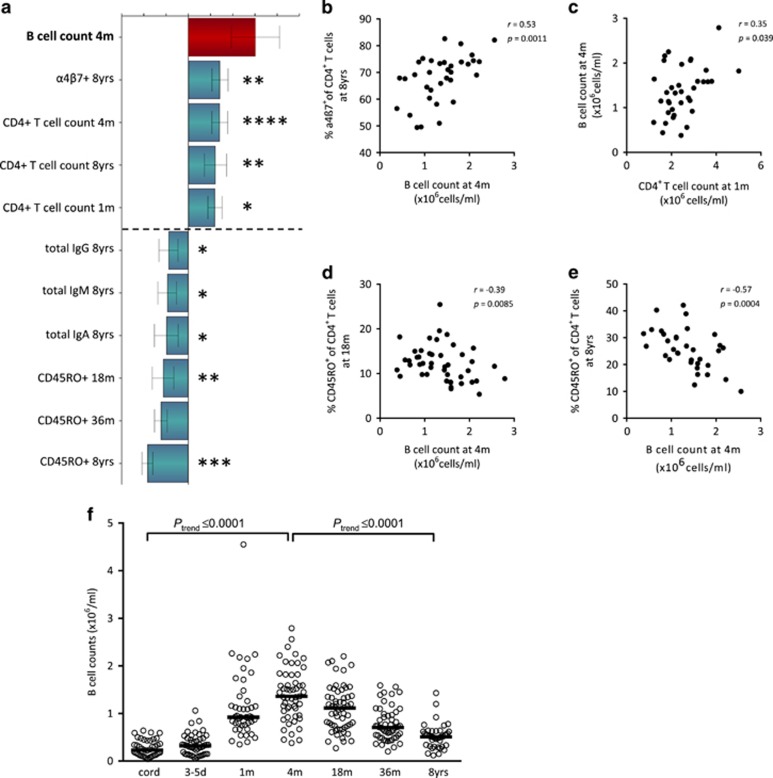
(**a**) OPLS-loading column plot displaying the associations between *Y*, that is, total numbers of B cells at 4 months of age, and *X* variables, that is, T- and B-cell variables over the first 9 years of life that characterize adaptive immune maturation. *X* variables with bars projected in the same direction as the respective *Y* variable are positively associated, whereas parameters in the opposite direction are inversely related to the *Y* variable. The larger the bar and smaller the error bar, the stronger and more certain is the contribution to the model. VIP values are used to discriminate between important and unimportant predictors for the overall model. The OPLS-loading column plot is based on *X* variables with VIP values⩾1.32. The quality of the OPLS analysis was based on the parameters R2, that is, how well the variation of the variables is explained by the model, and Q2, that is, how well a variable can be predicted by the model. R2Y=0.40; Q2=0.37. Statistical significant correlations (Spearman's rank correlation test) between *Y* and *X* variables are indicated by asterisks in the OPLS analysis. A *P-*value⩽0.05 was regarded as being statistically significant (*P⩽0.05; **P⩽0.01; ***P⩽0.001 and ****P⩽0.0001). (**b–e**) Representative univariate correlation plots from the OPLS analysis. (**f**) Total numbers of blood B cells within the lymphocyte gate at birth, at 3–5 days, at 1, 4, 18, 36 months and at 8 years of age. Horizontal bars indicate medians. ****⩽0.0001, analysis of variance post-test for linear trend. All data regarding B-cell numbers up to 36 months of age in the FARMFLORA study have been published previously by Lundell *et al.*^7^

It is not known whether the total numbers of blood lymphocytes are related to the maturation progress of the adaptive immune system in children. The negative correlations observed between total lymphocyte counts and vaccine-induced anti-DTP antibody titers (Figure 2) thus prompted us to investigate whether infantile total lymphocyte counts were related to the development of the adaptive immune system during the first 8 years of life. The final OPLS-loading column plots in Figures 3a and 4a display immune parameters that associate most strongly (variable importance for the projection (VIP) values ⩾1.2), positively or negatively, with total numbers of CD4^+^ T and B cells at 4 months of age, respectively.

The OPLS analysis in Figure 3a demonstrates that higher total numbers of CD4^+^ T cells at 4 months of age were associated with a higher proportion of α4β7^+^ T cells, previously shown to be more immature,^24^ and higher total numbers of B cells early in infancy as well as later in childhood. In contrast, higher total CD4^+^ T-cell counts at 4 months of age was associated with lower proportions of CD45RO^+^ memory T cells at 18 and 36 months and at 8 years of age (Figure 3a). These associations were all confirmed in univariate correlation analyses, as indicated with asterisks in Figure 3a and shown in Figures 3b and e. Similar multivariate association patterns were observed in OPLS analyses when CD4^+^ T-cell count in cord blood as well as at 1 month of age were analyzed as *Y* variables (Supplementary Figure 2). Total CD4^+^ T-cell counts in the blood from birth to 8 years of age are shown in Figure 3f.

Higher total numbers of B cells at 4 months of age were associated with a higher proportion of naïve α4β7^+^ T cells at 8 years, as well as with higher CD4^+^ T-cell counts over the first 8 years of life (Figures 4a–c). Finally, higher total numbers of B cells at 4 months were associated with lower proportions of CD45RO^+^ memory T cells at 18 and 36 months and at 8 years of age (Figures 4a, d and e) as well as with lower plasma levels of IgA, IgM and IgG at 8 years of age. Significant univariate correlation analyses are indicated with asterisks (Figure 4a). Total B-cell counts in the blood from birth to 8 years of age are shown in Figure 4f. In conclusion, our results suggest that a higher total number of lymphocytes in infancy may be a marker for a more immature/naïve adaptive immunity in general. Furthermore, the association between total numbers of lymphocytes and immune maturation/activation may be of relevance for vaccine responses since children with lower total counts of T and B cells in the blood in early infancy respond with higher anti-DTP antibody titers after vaccination (Figure 2).

### Post-vaccination DTP antibody titers correlate and were unrelated to gender, delivery mode and to dairy farm exposure

**Figure 5:**
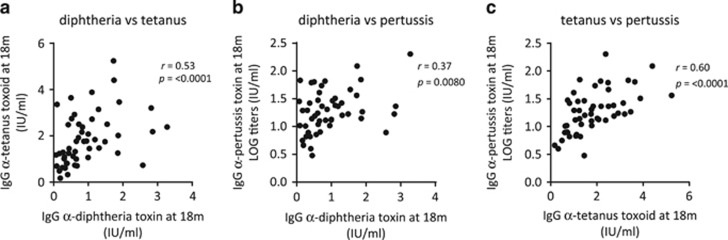
Correlation plots depicting post-vaccination anti-diphtheria toxin antibody titers in relation to (**a**) anti-tetanus toxoid antibody titers and (**b**) anti-pertussis toxin antibody titers, and (**c**) anti-tetanus toxoid antibody titers in relation to anti-pertussis toxin antibody titers at 18 months of age (Spearman's rank correlation test). A *P-*value⩽0.05 was regarded as being statistically significant.

Figures 5a and c show that levels of anti-DT antibodies (median 0.68, range 0.08–3.28 IU ml^−^^1^) correlated with both anti-TT (median 0.175, range 0.17–5.24 IU ml^−^^1^) and anti-PT (median 17.4, range 3–201.8 IU ml^−1^) antibody levels, and that anti-TT and PT antibody levels correlated strongly. Owing to the large inter-individual variations in anti-PT antibody levels, log values were used in Figures 5b and c. Two children presented with anti-DT antibody levels below correlate with protection, that is, <0.1 IU ml^−^^1^. One child presented with anti-PT antibody levels below those that indicate an immunization effect, that is, <4 IU ml^−^^1^. Moreover, gender, delivery mode and exposure to a dairy farm environment during early childhood were unrelated to vaccine-induced DTP antibody levels in this cohort (Table 1). Thus, children who respond with higher titers to one of the toxins are more likely to respond with higher titers to the other two toxins/toxoids included in the DTP vaccine.

## Discussion

To our knowledge, this is the first study to show the longitudinal relationship between postnatal adaptive immune maturation and DTP vaccine-induced antibody levels in young children. One principal finding was that children who presented with higher vaccine-induced antibody titers at 18 months of age had lower total numbers of circulating lymphocytes in early infancy. Another finding was that low total lymphocyte counts in infancy preceded a higher degree of T-cell maturation, represented by higher proportions of memory T cells later in childhood. Thus, our results suggest that low infantile total blood lymphocyte count, which represents a more mature/activated adaptive immunity, before and in close proximity to the first two DTP immunizations is related to higher anti-DTP antibody levels at 18 months of age.

Knowledge about the relationship between total numbers of lymphocytes in the circulation and the adaptive immune maturation progress in children has been lacking. In the present cohort, both total T- and B-cell counts increase immediately after birth, peak at about 4 months of life and subsequently decrease with age, as previously shown.^25^ A novel finding was that total CD4^+^ T-cell counts at 4 months of age displayed positive correlations with proportions of T cells with an immature phenotype, that is, α4β7-positive, and negative correlations with CD45RO^+^ memory T cells at several time points over the first 8 years of life. In addition, as a strong correlation was observed between total T- and B-cell counts at 4 months of age, total B-cell counts at this age displayed a similar multivariate association pattern with proportions of α4β7^+^ and CD45RO^+^ T cells. Total B-cell counts at 4 months of age further correlated negatively with total levels of plasma IgA, IgM and IgG at 8 years of age. Interestingly, a negative association could also be observed between total B-cell counts and vaccine-induced DTP antibody levels. We have previously shown that expression of the integrin α4β7 is linked to a naïve T-cell phenotype, as the proportion of α4β7^+^ T cells decrease in an age-dependent manner mirroring that of naïve CD45RA^+^ T cells.^2, 4^ In parallel, the fraction of CD45RO^+^ memory T cells increases during the first years in life.^4, 7^ Thus, a lower total lymphocyte count in infancy may be a marker for a more mature/activated T-cell immunity. However, the present study does not demonstrate that there is a direct causal relationship between low infantile lymphocyte counts and immune maturation/activation. Environmental, genetic and/or epigenetic factors may affect both numbers of lymphocytes and immune maturation/activation, for example, common childhood infections.

For the first time, we demonstrate that a low total lymphocyte count in the first 4 months of life is associated with higher DTP vaccine-induced antibody titers at 18 months of age. Since low total lymphocyte counts seem to represent an early biomarker of a more rapid postnatal activation of the CD4^+^ T cells, such activation may be beneficial for higher vaccine-induced DTP antibody responses. In line with this hypothesis, Turkish infants respond with higher DTP vaccine-induced antibody levels compared with Belgian infants.^26^ Further, Senegalese infants who received the same batch of DTP vaccine presented with post-vaccination titers in the same range as the Turkish infants.^26, 27^ Immunological mechanisms associated with these geographical differences are unclear, but a more traditional lifestyle is reflected by higher proportions of antigen-experienced lymphocytes in newborns.^28^ Also, neonates in areas with a more traditional lifestyle display a higher degree of activation of innate immunity genes.^29^ This could indicate 'trained innate immunity' also known as epigenetic reprogramming of innate immune cells and, as a consequence, heightened activation and phagocytic function of antigen-presenting cells.^30, 31^ Thus, children in less industrialized countries, that is, Turkey and Senegal, might have more mature/activated innate and adaptive immune functions at the time of DTP immunization compared with children in more industrialized countries such as Belgium.

In addition to the DTP vaccine, the children in the present cohort were also immunized with the measles–mumps–rubella vaccine that was initially given at 18 months of age with a booster at 6–8 years of age. It is likely that both the DTP and the measles–mumps–rubella vaccine influence maturation/activation of the adaptive immunity. However, as our main finding was that children who displayed higher vaccine-induced anti-DTP antibody levels at 18 months of age had lower numbers of total peripheral T cells already at birth and in the first month of life, at least these results could not have been influenced by vaccine-induced immune activation.

Although we and others have demonstrated that exposure to a farming environment affects the maturation of the innate and adaptive immune system, both pre- and postnatally,^16, 17, 18, 32, 33^ DTP titers did not differ between farmers' and non-farmers' children in the present study. Possibly, the divergence in immune maturation between these two groups of children, living in the same rural area, might not be distinct enough to affect the DTP antibody responses. An additional explanation might be the relatively small size of this study.

Opposed to our results regarding DTP vaccine responses and immune maturation, it has been shown that a more immature/naïve immune system was related to higher vaccine-specific antibody levels against yellow fever, that is, YF17D a live-attenuated virus vaccine.^34^ Young healthy adults in Switzerland responded with higher YF17D-induced antibody titers compared with vaccinees in Uganda, and the former group presented with lower proportions of memory T-cell subsets and higher fractions of naïve B cells before immunization.^34^ The reasons for these differences are still poorly understood. Yet, in view of the results from our study and the ones discussed above it appears as some vaccines might benefit from a more mature/activated immune system, whereas others are more effective if the immune system is more immature/naïve.

A limitation of this study is that only blood T- and B-cell subsets were examined, which may not be the most appropriate organ for this analysis. Advantages, however, is that our study is prospective in nature and that multivariate factor analysis was employed to investigate the longitudinal maturation/activation progress of the infantile adaptive immune system in relation to humoral DTP vaccine responses. This method enables discovery of patterns and trends, before proceeding with univariate analyses of *X* variables that contributed most to the respective models. The relatively small size of this study may be a limitation. Yet, the study was large enough to generate statistically significant results and permitted detailed and structured follow-up regarding immunological analyses.

In this study, we have for the first time shown how maturation/activation of adaptive immunity before and in close proximity to immunization is associated with magnitudes of DTP vaccine-induced antibody responses in 18-month-old children. A principal finding was that children who displayed higher vaccine-induced anti-DTP antibody levels at 18 months of age had lower total numbers of peripheral T and B cells in the first 4 months of life. Another novel finding was that low total numbers of infantile peripheral lymphocyte counts preceded a higher degree of memory T-cell conversion. Thus, our results suggest that children with a more mature/activated infantile adaptive immunity respond with higher vaccine-induced anti-DTP antibody levels. However, additional studies are required to identify specific factors that accelerate early-life adaptive immune maturation that appear to be beneficial for DTP vaccines responses in young children.

## Methods

### Subjects

Fifty-one Swedish infants from the prospective FARMFLORA study (*n*=64) whose parents agreed to take part in this part of the study were included.^18^ Twenty-one of the children were raised on small dairy farms, whereas 30 lived on the country-side in the same area, but not on farms. Ten percent (5/51) of the children were treated with antibiotics in the first 6 months of life. Blood samples were obtained from the umbilical cord and peripheral blood was sampled at 3–5 days and 1, 4, 18 and 36 months of age. Peripheral blood samples were also obtained from 43 children at ~8 years of age in the FARMFLORA follow-up study (median 8.1, range 6.4–9.4 years). All children received the combination vaccine diphtheria–tetanus–acellular pertussis–polio-*Haemophilus influenzae* type b (Pentavac, Sanofi Pasteur, Paris, France) at 3, 5 and 12 months and at 5–6 years of age. All parents provided written informed consent for their children, and the study was approved by the Human Research Ethics Committee of the Medical Faculty, University of Gothenburg, Sweden.

### Quantification of antibodies in plasma

Concentrations of IgG antibodies against DT, PT and TT, at 18 months of age were determined using in-house ELISA^35, 36^ or delayed fluorescense immunoassay,^37^ respectively, at the Public Health Agency of Sweden. For measuring anti-TT antibodies, TT were obtained from Statens Serum Institute (Copenhagen, Denmark), standard sera was obtained from Swiss Serum and Vaccine Institute (Bern, Switzerland) and lanthanide-labeled antigen (TTd Eu3+) was purchased from Wallac Oy (Turku, Finland). Specific anti-DT and TT antibody levels >0.1 IU ml^−1^ correlate with protection (Public Health Agency of Sweden). For PT there is no clear correlate with protection, but levels of specific anti-PT antibodies >4 IU ml^−1^ indicates an immunization effect (Public Health Agency of Sweden). Total IgM and IgA plasma levels in cord blood at 1, 4 and 18 months and 8 years of age, as well as total IgG levels in plasma at 18 months and 8 years of age, were determined by in-house ELISA as previously described in detail.^33^ Goat anti-human IgM, IgA and IgG were used as capture antibodies, and detection was performed by using horseradish peroxidase-conjugated goat anti-human IgM, IgA or IgG (all purchased from Jackson ImmunoResearch, Suffolk, England). Standards and polyclonal IgM, IgG or IgA from human plasma were obtained from Calbiochem (Darmstadt, Germany).

### Flow cytometry

Total numbers and phenotypic characterization of T and B cells in the FARMFLORA study was determined by flow cytometry as previously described in detail.^4, 7^ In brief, the anti-human monoclonal antibodies used are listed in Table 2. The PE-anti-human FOXP3-staining set was purchased from eBiosciences (San Diego, CA, USA) and the Cytofix/Cytoperm kit (BD Biosciences, Erembodegem, Belgium) was used for detection of CTLA-4. To assess the total numbers of T and B cells, the TruCOUNT assay was used according to the manufacturer's instructions (BD Bioscience). Lymphocytes were defined and further gated as low in the side scatter scale and high in CD45. Another dot plot was created to identify the beads with a FL1 versus FL2 plot where the beads were defined as having high FL1 and high FL2 properties. The following formula was used to get the numbers of lymphocytes per μl of blood: events of lymphocytes/events of beads × number of beads per TruCOUNT tube/blood volume. Finally, the total number of lymphocytes in each sample was multiplied with the fraction of CD4- or CD20-positive cells to get the total numbers of T and B cells. Thus, the TruCOUNT assay did not distinguish naïve from that of memory T and B cells. Yet, when analyzing the proportions of different T- and B-cell subsets both naïve and memory T and B cells were measured, as demonstrated by the gating strategy in Figure 1. Samples from 0 to 18 months of age were run in a FACS-Calibur (BD Biosciences) equipped with CellQuestPro software, whereas samples from the 8-year follow-up were run in a FACSCanto II (BD Biosciences) equipped with FACSDiva software. All samples were analyzed with FlowJo software (TreeStar, Ashland, OR, USA).

### Statistical analysis

Multivariate factor analysis (SIMCA software, Umetrics, Umeå, Sweden) can be used to explore large data sets to reveal patterns that may help to find relations between variables examined. To investigate associations between a *Y* variable (that is, the respective anti-DTP antibody titers or a selected immune parameter) and *X* variables (that is, the various T- and B-cell parameters) orthogonal projections to latent structures by means of partial least squares (OPLS) was implemented. The final OPLS-loading column plots presented in the result section are models based on *X* variables with VIP values ⩾1.1 (specified in the respective Figure legends). A representative full VIP plot for Figure 2a, which includes all immune parameters assessed and their contribution to the OPLS model is shown in Supplementary Figure 1. VIP values can be used to discriminate between important and unimportant predictors for the overall model. In the OPLS analyses, the importance of each *X* variable to the *Y* variable is represented by column bars. The larger the bar and smaller the error bar, the stronger and more certain is the contribution to the model. The quality of OPLS analyses is based on R2, how well the variation of the variables is explained by the model, and Q2, how well a variable can be predicted. For univariate analyses (GraphPad Prism; GraphPad Software, La Jolla, CA, USA), Spearman's rank correlation test (Figures 2d and i,3b and e and 4b and e) was performed exclusively on the *X* variables that contributed most to the respective multivariate OPLS models to avoid mass significance. Statistically significant correlations are indicated with asterisks in the respective OPLS plots. Analysis of variance post-test for linear trend was used in Figures 3f and 4f (horizontal bars indicate medians). *P⩽0.05, **P⩽0.01, ***P⩽0.001 and ****P⩽0.0001.

## Figures and Tables

**Table 1:** DTP vaccine-specific antibody titers at 18 months of age in relation to demographic data

		*Vaccine-specific antibody titers*					
		*IgG α-diphtheria toxin, IU ml^−1^*		*IgG α-tetanus toxoid, IU ml^−1^*		*IgG α-pertussis toxin, IU ml^−1^*	
	n	*Median (range)*	P*-value*^a^	*Median (range)*	P*-value*^a^	*Median (range)*	P*-value*^a^
*Gender*							
Girl	25	0.84 (0.08–3.3)	0.10	1.5 (0.33–3.5)	0.17	17 (3.0–200)	0.55
Boy	26	0.56 (0.09–1.7)		1.8 (0.17–5.2)		19 (4.6–120)	
							
*Delivery mode*							
Vaginal	43	0.66 (0.08–3.3)	0.62	1.8 (0.17–4.4)	0.75	19 (3.0–200)	0.37
C-section	8	1.1 (0.26–1.9)		1.8 (0.61–5.2)		15 (6.8–36)	
							
*Dairy farm*							
Yes	21	1.0 (0.08–2.9)	0.067	1.5 (0.17–3.5)	0.52	17 (4.6–70)	0.85
No	30	0.61 (0.09–3.3)		1.8 (0.33–5.2)		18 (3.0–200)	

a Statistical difference between the two groups of children, Mann–Whitney *U*-test.

**Table 2:** Antibodies used for characterization of T and B cells

*Monoclonal antibodies*	*Fluorochrome*	*Clone*	*Company*
CD4 (0–18 months)	PerCP	SK3	BD Biosciences, Erembodegem, Belgium
CD4 (8 years)	APC-H7	SK3	BD Biosciences
CD25	APC	2A3	BD Biosciences
CD45RO	PE	UCHL-1	BD Biosciences
FOXP3	PE	PCH101	eBioscience, San Diego, CA, USA
Biotin-CTLA-4	PE-streptavidin	BNI3	BD Biosciences
CD20 (0–18 months)	PerCP	L27	BD Biosciences
CD20 (8 years)	APC-H7	L27	BD Biosciences
CD5 (0–18 months)	APC	UCHT2	BD Biosciences
CD5 (8 years)	Brilliant Violet 421	UCHT2	BioLegend, San Diego, CA, USA
CD27	FITC	L128	BD Biosciences

Abbreviations: APC, allophycocyanin; FITC, fluorescein isothiocyanate; PE, phycoerythrin.
