# Supplementary material for: Earlier infantile immune maturation is related to higher DTP vaccine responses in children
Source: Clin Transl Immunology. 2016 Mar 11;5(3):e65–. doi: 10.1038/cti.2016.7 (PMC4815027; doi:10.1038/cti.2016.7)
Supplement: Supplementary Figure 1 [file cti20167x1.pdf]

VIP plot based on OPLS analysis including IgG anti-diphtheria at 18 months (Y) and all X-variables up to 18 months of age.

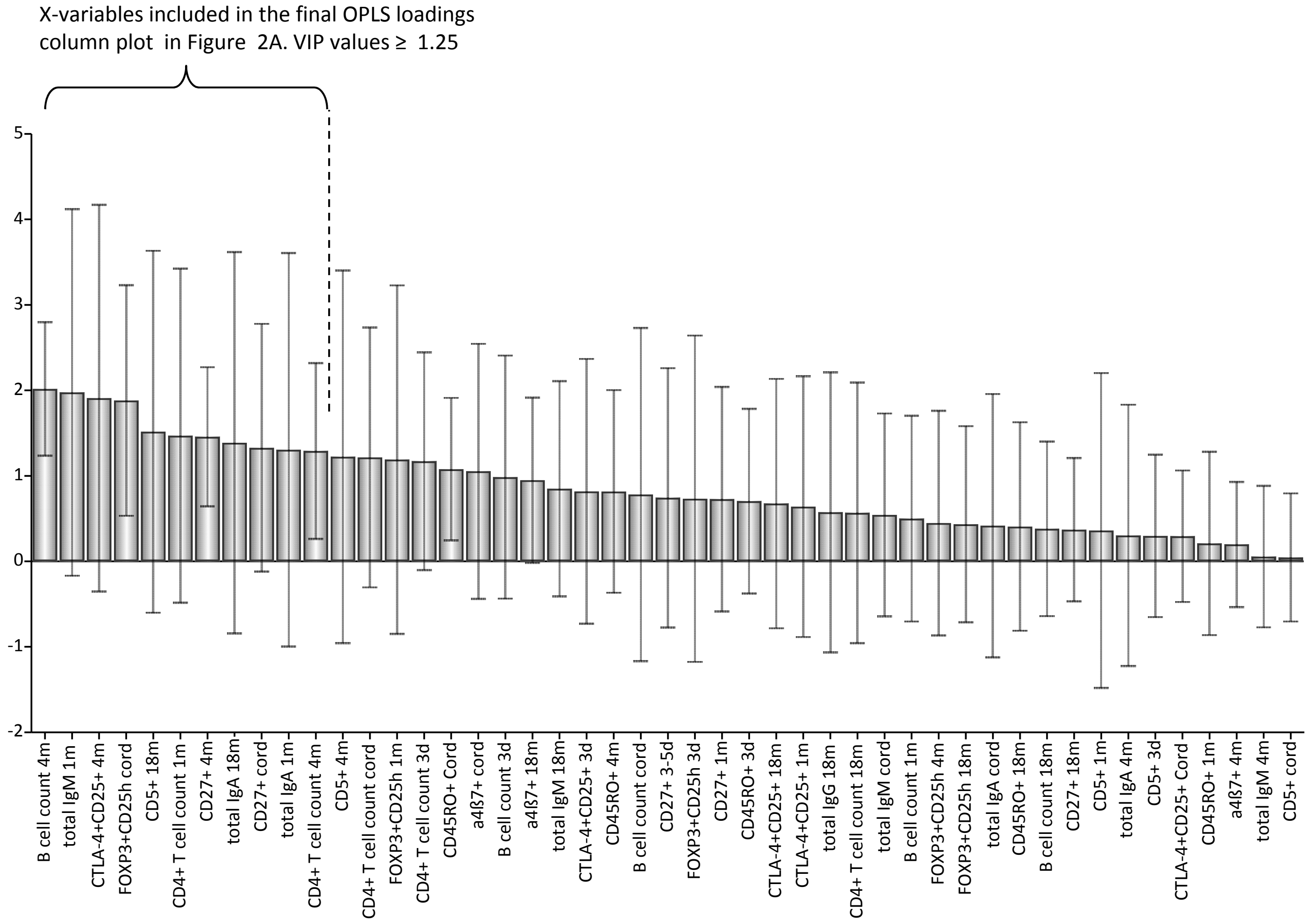

Figure S1. Representative variable importance for the projection (VIP) plot derived from the OPLS loadings column plot in figure 2A depicting associations between post-vaccination plasma titers of anti-diphtheria toxin antibodies at 18 months of age (Y-variable) and T- and B cell variables that characterize adaptive immune maturation (X-variables). VIP summarize the importance of the X-variables both to explain X and to correlate to Y. The plot is sorted to display the largest VIP to the left. Confidence intervals are derived from jack knifing.
