# Supplementary material for: Earlier infantile immune maturation is related to higher DTP vaccine responses in children
Source: Clin Transl Immunology. 2016 Mar 11;5(3):e65–. doi: 10.1038/cti.2016.7 (PMC4815027; doi:10.1038/cti.2016.7)
Supplement: Supplementary Figure 2 [file cti20167x2.pdf]

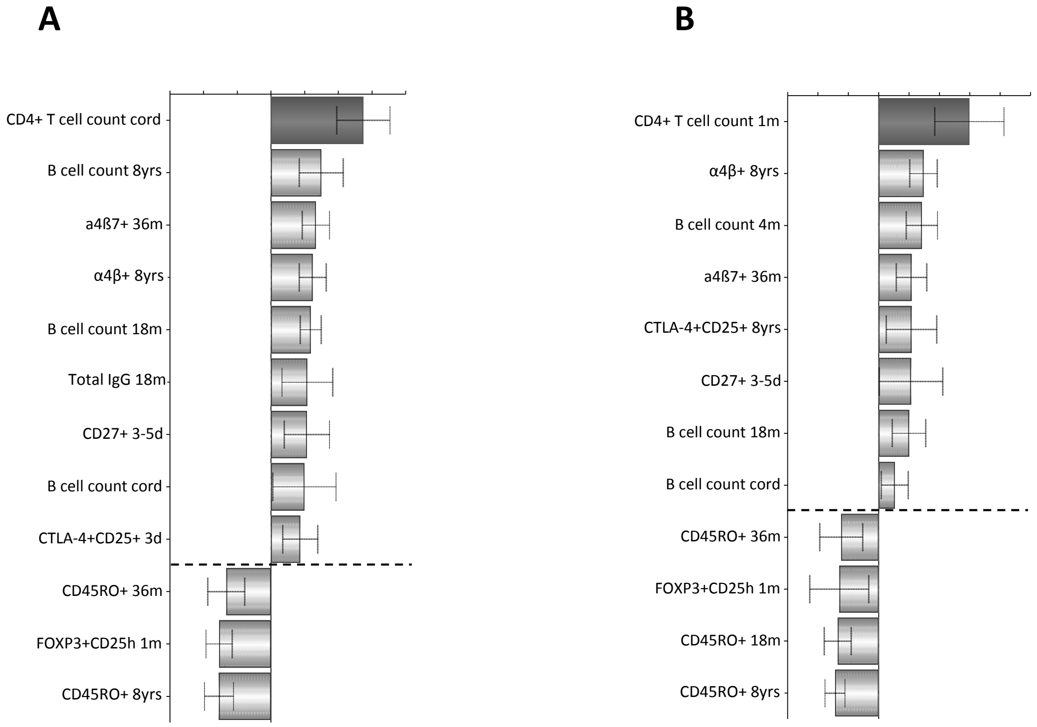


**Figure S2. (A)** OPLS loadings column plots displaying the associations between Y, i.e., numbers of $CD4^+$ T cells **(A)** in cord blood and **(B)** at 1 month of age and X-variables, i.e. T- and B cell variables that characterize adaptive immune maturation. X-variables with bars projected in the same direction as the respective Y-variable are positively associated, whereas parameters in the opposite direction are inversely related to the Y-variable. The larger the bar and smaller the error bar, the stronger and more certain is the contribution to the model. VIP values are used to discriminate between important and unimportant predictors for the overall model. The OPLS loadings column plots are based on X-variables with VIP values (A) $\geq$ 1.4 and (B) $\geq$ 1.45. The quality of the OPLS analyses was based on the parameters R2, i.e., how well the variation of the variables is explained by the respective model, and Q2, i.e., how well a variable can be predicted by the model. R2Y= **(A)** 0.57; **(B)** 0.46 and Q2 = **(A)** 0.37; **(B)** 0.38.
